# Incidence of Metabolic Syndrome and Its Risk Factors among Type 2 Diabetes Clinic Attenders in Isfahan, Iran

**DOI:** 10.5402/2012/167318

**Published:** 2012-03-15

**Authors:** Mohsen Janghorbani, Masoud Amini

**Affiliations:** ^1^Department of Epidemiology and Biostatistics, School of Public Health, Isfahan University of Medical Sciences, 8144503500 Isfahan, Iran; ^2^Isfahan Endocrine and Metabolism Research Center, Isfahan University of Medical Sciences, 8144503500 Isfahan, Iran

## Abstract

*Aim*. At present, little data exist about incidence and the risk factors associated with metabolic syndrome (MetS) in patients with type 2 diabetes mellitus (T2DM). The objectives of present study were to assess the incidence and risk factors of MetS in people with T2DM. *Methods*. During the mean (SD) follow-up period of 11.7 (4.8) years, 3,047 patients with T2DM and free of MetS at baseline have been examined to determine incidence and predictors of progression to MetS. A modified the National Cholesterol Education Program—Adult Treatment Panel III definition with body mass index (BMI) instead of waist circumference was used for the MetS. *Results*. The prevalence of MetS was 63.2% (95% CI: 62.3, 64.1). The incidence of MetS was 28.5 (95% CI: 26.8, 30.2) (25.9 men and 30.9 women) per 1,000 patient-years based on 35,677 patient-years of follow-up. Multivariate analysis revealed that higher BMI and education, lower HbA_1c_ and treatment with oral agent or insulin were associated with MetS. *Conclusion*. These are the first estimate of incidence and risk factors of MetS in patients with T2DM in Iran. These findings showed that the natural course of MetS is dynamic. The clinical management of patients with T2DM will contribute significantly to MetS prevention.

## 1. Introduction

Metabolic syndrome (MetS) is an important public health problem worldwide, and its prevalence is increasing [1, 2]. Patients with MetS are at higher risk for many long-term complications, including micro- and macro-vascular complications [2]. This is particularly relevant in patients with type 2 diabetes mellitus (T2DM), who are at even greater cardiovascular risk [3]. In fact, cardiovascular complications are the most common cause of morbidity and mortality in patients with T2DM [4]. The relationship between MetS and diabetes and cardiovascular disease is well established and consistent and has been examined in many different populations [3, 5, 6]. T2DM and cardiovascular disease have many risk factors in common, and many of these risk factors are highly correlated with one another [5, 7]. MetS is very common among patients with T2DM, using the Third Report of the National Cholesterol Education Program Adult Treatment Panel (NCEP/ATP III) definition; over 65% of patients with T2DM have MetS [8]. This is much higher prevalence than in comparable general populations [9, 10]. The higher prevalence of MetS in patients with T2DM may be explained by medication-, disease-, and lifestyle-related factors. Limited information is available about the incidence of the MetS and its risk factors in patients with T2DM and none in Iran.

Accurate information regarding the incidence of MetS and associated risk factors in people with T2DM is important to get a better understanding of natural course of metabolic and cardiovascular risk in a non-preselected cohort of diabetic patients in routine practice.

This study, therefore, used routinely collected data from a clinical information system for diabetes at Isfahan Endocrine and Metabolism Research Center, Iran, to estimate the incidence of MetS and to identify its risk factors in a large sample of diabetic patients receiving routine care.

## 2. Patients and Methods 

### 2.1. Participants and Data Collection

The recruitment methods and examination procedures of the Isfahan Endocrine and Metabolism Research Center outpatient clinics have been described before [11, 12]. In summary, clinical data are collected for all consecutive patients at the first attendance and at review consultations (usually annually) using standard encounter forms. These include an examination of ocular fundus and lens, the limbs, and blood pressure (BP); measurement of height, weight, fasting plasma glucose (FPG), glycosylated haemoglobin (HbA_1c_), urine protein, triglyceride, cholesterol, and serum creatinine levels. The clinician compiles a list of problems and smoking is reported via a questionnaire completed by the patients on demography, family history, and smoking. 

 Generally, newly diagnosed patients are referred to qualified nutritionists for evaluation; if necessary, a lifestyle and weight management program is recommend. All newly diagnosed patients attend weight-related health education classes free of charge.

### 2.2. Participants

Using routinely collected data from a clinical information system at Isfahan Endocrine and Metabolism Research Centre, Iran, we performed a retrospective longitudinal, observational study. The study population consisted of all prevalent cases of T2DM, and all patients were diagnosed during the study period. Between 1992 and 2009, a total of 11,281 patients with T2DM were registered in the system. However, this study uses data only for 3,047 of these patients, that is, 1,461 (47.9%) men and 1,586 (52.1%) women who had at least one subsequent review since registration and who were free of MetS at baseline. The physician defined the type of diabetes according to the American Diabetes Association criteria [13]. 

The study conformed to the Declaration of Helsinki. Institutional ethical committee approval was granted, and an informed consent was signed by each patient.

### 2.3. Ascertainment of MetS

A minimally modified NCEP/ATP III [14] definition with body mass index (BMI) instead of waist circumference was used for the MetS by the presence of three or more of the following abnormalities: blood pressure ≥ 130/85 mmHg or a history of hypertension and current use of antihypertensive treatment; BMI ≥ 25 kg/m^2^; serum triglyceride ≥ 150 mg/dL (≥1.7 mmol/L); high-density lipoprotein cholesterol (HDL; <40 mg/dL (<0.9 mmol/L) for men and < 50 mg/dL (<1.0 mmol/L) for women); known diabetes mellitus. BMI ≥ 25 kg/m^2^ was used instead since waist circumference was not available. In some other studies, BMI has been adopted instead of waist circumference for analysis of MetS [5, 8, 15, 16].

### 2.4. Procedures

Predictors of progression to MetS were assessed using the following data from the patient's registration consultation: gender, age at diagnosis (i.e., at the time this was first recorded by a physician on the participant's chart), current age (at the time of examination), educational level, duration of diabetes (the time between diagnosis and the baseline examination), BMI (weight/height^2^ [kg/m^2^]), smoking status (never, current), HbA_1c_ (measured by ion-exchange chromatography), FPG (measured by glucose oxidase method; Clinical Chemistry Analyzer Liasys, Italy), proteinuria (measured by precipitation with 3% sulfosalicylic acid and determination of turbidity by measuring absorbance at a wavelength of 550 nm with a spectrophotometer), serum creatinine, triglyceride, cholesterol, HDL (measured using standardized procedures), and low-density lipoprotein cholesterol (LDL; calculated by the Friedewald Equation [17]) levels 

 Height and weight were measured using standard apparatus, with the subjects in light clothes and without shoes. Weight was measured to the nearest 0.1 kg on a calibrated beam scale. Height was measured to the nearest 0.5 cm and assessed at baseline only. A physician measured the systolic and diastolic BPs of the participants (after they had been seated for 10 minutes) using a mercury sphygmomanometer and standard techniques. All clinical and laboratory measurements at baseline and followups were made using the same standardized protocol.

### 2.5. Determination of MetS Incidence

Incidence of MetS was expressed as the number of cases of MetS per 1,000 patient-years of followup. As the relevant period was considered, the date of completion of the baseline examination between 1992 and 2009 until the either (i) occurrence of MetS, (ii) the date of the last completed followup, (iii) death, or (iv) end of followup on December 31, 2009, whichever came first.

### 2.6. Statistical Analysis

The statistical methods used included the Student's *t*-test; chi-square test, analysis of variance (ANOVA) or Kruskal-Wallis tests for normally or nonnormally distributed continuous variables, respectively and Cox's proportional hazards model. Univariate and multivariate Cox's proportional hazards models were fitted to identify predictors of new-onset MetS using SPSS version 18 for Windows (SPSS Inc., Chicago, IL, USA). All the significant variables in the bivariate analysis were included as independent variables in a multivariate Cox's proportional hazards models. Adjustment for age was examined in separate models. Age-adjusted means were calculated and compared using general linear models. All tests for statistical significance were two-tailed, confidence intervals (CIs) were set at 95%, and *P* < 0.05 was considered significant.

## 3. Results

### 3.1. Subject Characteristics

Differences in distribution of several risk factors among 1,461 men and 1,586 women are shown in Table 1. Women had slightly lower creatinine, were less likely to be smokers, and were younger at registration and had lower dyslipidemia than men. Men had lower BMI, total cholesterol, HDL, and LDL cholesterol than women. The mean (SD) BMI was 24.0 (3.3) kg/m^2^ for men and 25.7 (4.3) kg/m^2^ for women. The prevalence of overweight (BMI ≥ 25) was 25.1% (95% CI: 22.7 and 27.4%) in men, and 45.1% (95% CI: 42.5 and 47.8%) in women. Only 8.1% (95% CI: 6.7 and 9.8%) of men, and 5.10% (95% CI: 4.0 and 6.4%) of women were underweight (BMI ≤20). The majority of patients were on oral agent (61.0%), and 22.6% of the sample was on diet and exercise. A total of 16.4% of patients were on insulin treatment. 

### 3.2. Prevalence

As defined by the modified NCEP/ATP III criteria, of the 11,281 patients with T2DM, 7,132 (2,584 men and 4,548 women) had MetS. Overall prevalence of MetS was 63.2% (95% CI: 62.3 and 64.1). Prevalence rates were higher in women (68.8% (95% CI: 67.7 and 69.9) than men (55.3% (95% CI: 53.9 and 56.8). The prevalence of MetS increased with age. Of the 1,412 patients who were insulin-treated, 772 had MetS, giving a prevalence of 54.7% (95% CI: 52.1 and 57.3). This was lower than the prevalence rates seen for noninsulin-treated, 66.3% (95% CI: 65.3 and 67.2). 

Most of diabetic patients had three components of the syndrome (36.4%), 23.4% had four, and 3.4% had five components. Only 9.2% of the diabetic patients were free from any other components of the syndrome, and 27.6% had one more component.

### 3.3. Incidence

Of the 3,047 participants without MetS, 1017 (33.4%) (446 men and 571 women) developed MetS in 35,677 (17,205 men and 18,472 women) patient-years of followup. The overall incidence of subsequent MetS was 28.5 (95% CI: 26.8 and 30.2) per 1,000 patient-years. Incidence rates were higher in women (30.9 (95% CI: 28.4 and 33.4) per 1,000 patient-years) than men (25.9 (95% CI: 23.5 and 28.3). This difference was statistically significant (*P* < 0.01). Of the 501 patients who were insulin-treated, 174 subsequently developed MetS, giving an incidence of 30.1 per 1,000 patient-years (95% CI: 25.7 and 34.5). This was similar to the incidence rate seen for oral agent-treated, 29.4 per 1,000 patient-years (95% CI: 27.2 and 31.7), but slightly higher than the incidence rate seen for exercise- and diet-treated 24.9 (95% CI: 21.0 and 28.2).

### 3.4. Risk Factors

Table 2 shows the group means (SE) and proportions for those who did and did not develop MetS. Those who developed MetS were more women and had higher weight, BMI, cholesterol, number of follow-up visits, and proportion of obesity at baseline. Those who did not develope MetS were more likely to be smokers and had slightly higher follow-up period, height, and educational level than those who developed MetS. 

A univariate analysis (Table 3) showed that age, gender, lower HbA_1c_, BP, triglyceride, oral antihyperglycemic therapy, overweight and obesity, and no smoking were significantly associated with the risk of developing MetS. Age-adjusted Cox regression coefficient among those free of MetS at registration showed that significant risk factors for developing MetS were shorter duration of diabetes, lower FPG, cholesterol, triglyceride, creatinine, no smoking, higher education, oral agent or insulin treatment, and overweight and obesity. 

The incidence of MetS was also analyzed with multivariate model. Cox's proportional hazards model showed that higher BMI (RR 1.04; 95% CI: 1.03 and 1.05), higher education (RR 1.48; 95% CI: 1.29 and 1.69), and lower HbA_1c_ (RR 0.90; 95% CI: 0.88 and 0.93), and treatment with insulin (RR 1.22; 95% CI: 1.10 and 1.35) or oral agent (RR 1.25; 95% CI: 1.09, 1.43) at baseline significantly predicted the onset of MetS after mean 11.7 years. No other variables were significant.

## 4. Discussion

In this follow-up study of 3,047 participants, the natural course of MetS in patients with T2DM described. The incidence of MetS was 28.5 per 1,000 patient-years over an average followup of 11.7 years. The incidence rates were 25.9 per 1,000 patient-years in men and 30.9 in women. It seems that the higher BMI, educational level, lower HbA_1c_, and treatment with insulin or oral agent at baseline, the higher the risk of progression to MetS. To our knowledge, little research has been done to estimate incidence of MetS in patients with T2DM. Therefore, we cannot compare our findings with those of other studies. Incidence and prevalence rates of MetS in general populations in various studies from around the world show considerable variation [18, 19]. Estimates of incidence and prevalence of MetS will depend upon the methodological factors, the definition of the MetS used, and the composition of the community examined by age, gender, ethnicity, and social class, making comparisons between studies of limited values. Several cross-sectional evaluations conducted at different moments and in different populations show considerable variation. The prevalence of MetS in people with T2DM of 63.2% as reported in this study is much higher than the values reported in general populations [9, 20–22] and similar to the studies on T2DM from other diabetic populations [23–25].

The incidence rate of MetS that we report in the present study is lower than that reported in low-risk population studies carried out in the Japan [26], Europe [27], and North America [28–30] may be due to routine diabetes care. Patients with higher BP and dyslipidemia were treated during routine care. Baltimore Longitudinal Study of Aging reported an incidence of 25.5% in men and 14.8% in women after an average followup of 6 years [28]. The Insulin Resistance Atherosclerosis Study reported an incidence of 17.1% in men and 20.9% in women after a follow-up period of 5 years [29]. Longitudinal study of Japanese men ages 35 to 59 reported that incidence of MetS was 3.6 per 100 person-years [26]. Longitudinal study of Korean male workers aging from 30 to 39 reported that incidence of MetS was 77 per 1,000 person-years [31]. Another study from an urban area of Portugal reported an incidence of 47/1,000 person-years, similar in men and women [27]. But it is higher than that reported in the San Antonio Heart Study. It showed a 15% incidence of MetS in men and a 17% in women after 8 years of followup [30]. The threshold values used to define MetS criteria were higher than those indicated by NCEP/ATP III for lipids (triglyceride ≥200 mg/dL, HDL <35 mg/dL in men and <45 mg/dL in women) and BP (BP ≥140/90 mmHg for systolic and diastolic, resp.). However, our findings indicate that patients with T2DM appearing at higher risk for developing MetS do not actually develop it. A possible explanation is that these patients may adjust their habits toward healthier lifestyle, besides receiving appropriate treatment for hypertension and hyperlipidemia. 

Several risk factors predicted the incidence MetS in our study. Univariate analysis (Table 3) shows an expected pattern of association for many variables with the development of MetS. Participants who subsequently developed MetS had greater obesity, higher triglycerides, lower smoking, and higher proportion of insulin- or oral agent-treated at baseline than those who did not develop the MetS. In multivariate analysis, fewer remain independently associated. The people with T2DM who were insulin- or oral agent-treated were at higher risk of MetS than those who treated with diet and exercise. Insulin or oral agent treatment may indicate a more severe disease process. A higher incidence of MetS among insulin- or oral agent-treated patients could be attributable to their longer duration of diabetes, younger age at onset, and poorer metabolic control than in noninsulin-treated diabetes. Other longitudinal studies documented the pivotal role of obesity in the pathogenesis of MetS in different populations, although the definition of MetS adopted varied from study to study [28, 30]. 

The lack of correlation between incident MetS and elevated BP at baseline in our patients is not surprising. In fact, this sort of “dissociation” may affect the decision-making process in a clinical setting tailored for preventing MetS and cardiovascular events. 

The role of gender as a risk factor for MetS remains unsettled. There have been conflicting reports about the relationship between gender and MetS incidence in general populations; in some studies MetS incidence was higher in women [27, 30]; whereas in other studies it was higher in men [32, 33]. Similar to our results, some other cohorts from different ethnic background reported no significant differences regarding gender [29, 34, 35]. 

The higher MetS incidence found in lower values of HbA_1_ was probably related to this fact that patients with higher values of HbA_1_ probably are more deficient in insulin and less insulin resistant, which could have reduced their probability to present MetS. However, this warrants further study. 

Some limitations warrant consideration. The Isfahan clinical information system for diabetes provides one of the largest clinic-based data sets of its kind in the developing world. Although we have not carried out any special studies of the validity or reliability of data for this analysis, a clerk was employed to check consistency and, where possible, to ensure completeness of data. Previous studies show that these patients are a representative sample of known diabetic patients of Isfahan [36]. Our experience with other parts of the data set gives us some confidence that data quality is sufficient for this type of study, and that our results provide useful additional evidence on the incidence of and risk factors for MetS. The study was clinic, rather than population-based, and so may not contain a clinical spectrum representative of diabetic patients in the community. Many patients requiring only oral or dietary treatment may never attend the clinic. Clinic-based estimates of the incidence or prevalence of complications are most likely to be affected by referral patterns. Selection bias is less likely to affect incidence rates and associations between risk factors and complications as investigated in this study. The study was performed according to the modified NCEP/ATP III criteria [14]. We used BMI instead of waist circumference due to unavailability of data regarding waist circumference in our database. The central pattern of distribution, with its higher weighting of waist circumference, is associated with more insulin resistance than is the peripheral pattern of distribution [37, 38]. Nevertheless, although waist measurement is easy and not time-consuming, waist is not routinely measured in clinical practice. A number of studies have also shown that BMI is as effective as waist circumference for predicting the development of T2DM and other metabolic disturbances [5, 8, 15, 16]. In addition, the Japan Society for the Study of Obesity has reported that BMI can estimate visceral fat measured by computed tomography as robustly as waist circumference, and that obesity-related complications increase for a BMI of 25 [39]. An additional limitation of the present study is represented by the lack of information on the effect of medications (lipids lowering and antihypertensive) on the trajectory of MetS components. Despite the above limitations, the findings here add to our understanding of the incidence, prevalence, risk factors, and the natural course of MetS in patients with T2DM in Iran. Furthermore, this study provides new data from Iran, a developing country that has been underrepresented in past studies. 

In conclusion, this longitudinal study provides information on the high prevalence but low incidence rate of MetS in patients with T2DM in Isfahan, Iran. Our study shows that in routine practice the natural course of MetS in patients with T2DM is dynamic. The clinical management of patients with T2DM will contribute significantly to MetS prevention. 

## Figures and Tables

**Table 1:** Age and age-adjusted means (SE) and proportions of selected characteristics among 1461 men and 1586 women.

Variables	Men	Women
	Mean (SE)	Mean (SE)
Age at registration (yr.)	52.3 (0.28)	48.7 (0.27)***
Duration of diabetes (yr.)	6.5 (0.16)	6.2 (0.15)
Age at diagnosis (yr.)	43.9 (0.16)	44.2 (0.15)
BMI (Kg/m^2^)	24.0 (0.11)	25.7 (0.10)***
Systolic BP (mmHg)	116.1 (0.37)	115.1 (0.35)*
Diastolic BP (mmHg)	72.0 (0.27)	71.8 (0.25)
Fasting blood glucose (mg/dL)	203.4 (2.11)	199.0 (2.02)
HbA1 (%)	8.8 (0.10)	8.5 (0.10)*
Creatinine (mg/dL)	1.04 (0.03)	0.88 (0.02)***
Triglyceride (mg/dL)	164.2 (3.21)	156.0 (3.10)
Cholesterol (mg/dL)	201.0 (1.25)	213.1 (1.20)***
HDL Cholesterol (mg/dL)	46.9 (0.66)	55.8 (0.66)***
LDL Cholesterol (mg/dL)	118.4 (2.32)	126.6 (2.34)*

	%	%

Obesity (BMI ≥30)		
Currentsmoker	32.5	2.6***
Dyslipidemia^†^	37.1	32.6*
Therapeutic regimen		
Diet	21.7	23.4*
Oral agent	61.5	60.6
Insulin	16.9	16.1
Education		
Primary or below	46.7	72.2***
Secondary	32.3	22.2
Matriculation or above	21.0	5.6

*P < 0.05, **P < 0.01, and ***P < 0.001. BP: blood pressure; HDL: high density lipoprotein cholesterol; LDL: low-density lipoprotein cholesterol. ^†^Dyslipidemia: triglyceride ≥150 mg/dL (≥1.7 mmol/L) or HDL cholesterol < 40 mg/dL (<0.9 mmol/L) in men or <50 mg/dL (<1.0 mmol/L) in women.

**Table 2:** Age and age-adjusted means (SE) and proportions of selected baseline characteristics between 1017 patients with type 2 diabetes who did and 2030 who did not develop metabolic syndrome (MetS).

Variables	Developed MetS	Not developed MetS	Difference (95% CI)
	Mean (SE)	Mean (SE)	
Age at registration (yr.)	50.8 (0.34)	50.2 (0.24)	0.6 (−0.23, 1.43)
Duration of diabetes (yr.)	6.2 (0.18)	6.4 (0.13)	−0.2 (−0.62, 0.32)
Age at diagnosis (yr.)	44.3 (0.18)	44.0 (0.13)	0.3 (0.00, 1.59)
Followup (yr.)	11.6 (0.15)	12.0 (0.11)	−0.4 (−1.13, −0.39)***
Number of follow-up visits	15.6 (0.41)	9.6 (0.29)	6.0 (5.01, 6.99)***
Height (cm)	159.8 (0.29)	161.5 (0.21)	−1.7 (−2.41, −0.99)***
Weight (kg)	66.8 (0.36)	64.0 (0.25)	2.8 (1.68, 3.73)***
BMI (Kg/m^2^)	26.0 (0.12)	24.3 (0.09)	1.7 (1.40, 2.00)***
Systolic BP (mmHg)	116.1 (0.43)	115.3 (0.31)	0.8 (−0.30, 1.90)
Diastolic BP (mmHg)	72.4 (0.31)	71.6 (0.23)	0.8 (0.00, 1.56)
Fasting glucose (mg/dL)	203.5 (2.49)	200.0 (1.78)	3.5 (−2.50, 9.50)
HbA_1c_ (%)	8.7 (0.11)	8.6 (0.09)	0.1 (−0.18, 0.38)
Creatinine (mg/dL)	0.94 (0.03)	0.97 (0.02)	−0.03 (−0.10, 0.04)
Triglyceride (mg/dL)	162.8 (3.77)	158.3 (2.76)	4.5 (−4.65, 13.70)
Cholesterol (mg/dL)	210.7 (1.48)	205.5 (1.07)	5.2 (−1.61, −8.79)**
HDL cholesterol (mg/dL)	51.0 (0.74)	51.6 (0.68)	−0.6 (−2.58, 1.38)
LDL cholesterol (mg/dL)	124.0 (2.44)	121.2 (2.24)	2.8 (−3.51, 9.51)
	%	%	
Men	43.9	50.0	−6.1 (−9.9, −2.4)**
Obesity (BMI ≥30)	15.7	8.4	7.3 (4.7, 10.00)***
Dyslipidemia^†^	33.6	35.4	−1.8 (−5.4, 1.9)
Currentsmoker	12.9	19.5	−6.6 (−9.71, −3.61)***
Therapeutic regimen			
Diet	20.7	23.5	−2.8 (−5.81, 0.38)
Oral agent	62.1	60.5	1.6 (−1.97, 5.35)
Insulin	17.1	16.1	1.0 (−1.79, 3.84)
Education			
Primary or below	64.2	57.6	6.6 (2.82, 10.30)**
Secondary	25.4	28.0	−2.6 (−5.92, 0.89)
Matriculation or above	10.3	14.4	−4.1 (−6.54, −1.57)**

*P < 0.05, **P < 0.01, and ***P < 0.001. CI: Confidence interval; BP: blood pressure; HDL: high-density lipoprotein cholesterol; LDL: low-density lipoprotein cholesterol. The difference in the mean or percentage of the variables between MetS and no MetS. ^†^Dyslipidemia: triglyceride ≥150 mg/dL (≥1.7 mmol/L) or HDL cholesterol < 40 mg/dL (<0.9 mmol/L) in men or <50 mg/dL (<1.0 mmol/L) in women.

**Table 3:** Incidence rates and relative risks (RR) for metabolic syndrome by baseline variables.

Variables	At risk	Case	Person-year	Incidence/1000 person-year	Crude RR	Age-adjusted RR (95% CI)^†^
	(number)	(number)			(95% CI)	
All	3,047	1017	35677	28.5	—	—
Gender						
Men	1,461	446	17205	25.9	1.00	1.00
Women	1,586	571	18472	30.9	1.19 (1.06, 1.35)**	0.99 (0.84, 1.70)
Age (yr.)						
<40	485	133	5552	23.9	1.00	—
40–49	946	321	10603	30.3	1.27 (1.04, 1.54)**	—
50–59	946	351	10732	32.7	1.37 (1.12, 1.66)**	—
60–69	508	165	6700	24.6	1.03 (0.82, 1.29)	—
≥70	160	47	2134	22.0	0.92 (0.66, 1.28)	—
Age at diagnosis (yr.)						
<30	198	58	2333	24.9	1.00	—
30–59	2584	874	30116	29.0	1.16 (0.90, 1.52)	—
≥60	251	80	3126	25.6	1.03 (0.74, 1.44)	—
Duration of diabetes (yr.)						
<5	1,551	522	17101	30.5	1.00	1.00
5–7	540	173	6450	26.8	0.88 (0.74, 1.04)	0.85 (0.77, 0.94)**
8–11	422	143	5104	28.0	0.92 (0.77, 1.10)	0.79 (0.71, 0.88)***
≥12	522	174	6927	25.1	0.82 (0.70, 0.98)	0.71 (0.65, 0.80)***
Fasting glucose (mg/dL)						
<100	139	51	1428	35.7	1.00	1.00
≥100	2809	951	33258	28.6	0.80 (0.61, 1.06)	0.78 (0.66, 0.93)**
HbA_1c_ (%)						
<6.5	258	99	1407	70.4	1.00	1.00
≥6.5	1,034	423	8667	48.8	0.69 (0.56, 0.86)*	0.51 (0.45, 0.59)***
Systolic BP (mmHg)						
<130	2556	919	28892	31.8	1.00	1.00
≥130	348	96	4410	21.8	0.69 (0.56, 0.84)**	0.90 (0.80, 1.01)
Diastolic BP (mmHg)						
<85	2709	966	30796	31.4	1.00	1.00
≥85	189	49	2413	20.3	0.65 (0.49, 0.86)**	0.88 (0.76, 1.02)
Cholesterol (mg/dL)						
<200	1332	429	14494	29.6	1.00	1.00
200–219	512	182	6038	30.1	1.02 (0.86, 1.21)	0.84 (0.76, 0.93)
>220	1010	373	12892	28.9	0.98 (0.85, 1.12)	0.75 (0.69, 0.81)***
HDL (mg/dL)						
Men ≥40 & women ≥50	438	210	3883	54.0	1.00	1.00
Men <40 & women <50	106	39	989	39.4	0.73 (0.52, 1.02)	0.85 (0.68, 1.05)
LDL (mg/dL)						
<100	144	57	1226	46.5	1.00	1.00
≥100	388	186	3533	52.6	1.13 (0.85, 1.51)	0.86 (0.71, 1.05)
Triglyceride (mg/dL)						
<150	1951	693	21754	31.9	1.00	1.00
≥150	878	292	11276	25.9	0.81 (0.71, 0.93)**	0.73 (0.67, 0.79)***
BMI (kg/m^2^)						
<25	1688	511	20077	25.5	1.00	1.00
25–29.9	644	300	6739	44.5	1.75 (1.52, 2.01)***	1.24 (1.12, 1.35)***
≥30	293	153	2803	54.6	2.14 (1.80, 2.56)***	1.47 (1.29, 1.67)***
Smoking						
Nonsmoker	1979	677	25960	26.1	1.00	1.00
Currentsmoker	416	100	5820	17.2	0.66 (0.54, 0.81)***	0.87 (0.74, 0.98)*
Education						
Primary or below	1718	621	21273	29.2	1.00	1.00
Secondary	778	246	8142	30.2	1.03 (0.89, 1.20)	1.25 (1.14, 1.36)***
Matriculation or above	374	100	3782	26.4	0.90 (0.74, 1.12)	1.37 (1.23, 1.54)***
Creatinine (mg/dL)						
≤1.5	2159	823	22669	36.3	1.00	1.00
>1.5	61	21	841	25.0	0.69 (0.45, 1.05)	0.60 (0.47, 0.77)***
Therapeutic regimen						
Diet	687	211	8471	24.9	1.00	1.00
Oral agent	1860	632	21480	29.4	1.18 (1.01, 1.38)***	1.25 (1.11, 1.40)***
Insulin	500	174	5777	30.1	1.21 (0.99, 1.49)	1.20 (1.10, 1.32)***

Total number of patient-years and at risk is not the same for each variable because of missing values. ^†^Relative risks (with 95% CI) calculated by Cox's proportional hazards model. CI: Confidence interval; RR: relative risk; BP: blood pressure; HDL: high-density lipoprotein cholesterol; LDL: low-density lipoprotein cholesterol. *P < 0.5, **P < 0.01, and ***P < 0.001.
